# Prediagnostic detection of mesothelioma by circulating calretinin and mesothelin – a case-control comparison nested into a prospective cohort of asbestos-exposed workers

**DOI:** 10.1038/s41598-018-32315-3

**Published:** 2018-09-25

**Authors:** Georg Johnen, Katarzyna Burek, Irina Raiko, Katharina Wichert, Beate Pesch, Daniel G. Weber, Martin Lehnert, Swaantje Casjens, Olaf Hagemeyer, Dirk Taeger, Thomas Brüning, MoMar Study Group, Alexander Brik, Judith Delbanco, Bettina Dumont, Jan Gleichenhagen, Ulrike Gross, Heike Heimann, Evelyn Heinze, Monika Kobek, Claudia Lechtenfeld, Swetlana Meier, Carmen Meinig, Simone Naumann, Simone Putzke, Hans-Peter Rihs, Peter Rozynek, Sandra Schonefeld, Jens Schreiber, Katja Szafranski, Thorsten Wiethege, Sandra Zilch-Schöneweis

**Affiliations:** Institute for Prevention and Occupational Medicine of the German Social Accident Insurance, Institute of the Ruhr University Bochum (IPA), Bochum, Germany

## Abstract

Malignant mesothelioma (MM) is strongly associated with a previous asbestos exposure. To improve timely detection of MM in asbestos workers, better screening tools – like minimally-invasive biomarkers – are desirable. Between 2008 and 2018 2,769 patients with benign asbestos-related diseases were recruited to participate in annual screens. Using a nested case-control design the protein markers calretinin and mesothelin were determined by enzyme-linked immunosorbent assays in prediagnostic plasma samples of 34 MM cases as well as 136 matched controls from the cohort. Conditional on a pre-defined specificity of 98% for calretinin and 99% for mesothelin the markers reached individual sensitivities of 31% and 23%, respectively, when including the incident cases with samples taken between one and 15 months before diagnosis. The combination of both markers increased the sensitivity to 46% at 98% specificity. Marker complementation increased with earlier sampling. The marker combination improves the sensitivity of the individual markers, indicating a useful complementation and suggesting that additional markers may further improve the performance. This is the first prospective cohort study to evaluate a detection of MM by calretinin and its combination with mesothelin up to about a year before clinical diagnosis. Whether an earlier diagnosis will result in reduced mortality has yet to be demonstrated.

## Introduction

Asbestos is still used in many countries despite being classified as a human carcinogen by the International Agency for Research on Cancer (IARC) in 1977^1,2^. Because of the continued use of asbestos and the long latency period ranging from less than ten to more than 70 years, asbestos-related cancers like malignant mesothelioma (MM) remain a global health issue^3,4^. Estimates of the global annual number of MM deaths are in the range of 32,000 to 59,000^5^. In Germany, more than 1,600 new MM cases are diagnosed annually and over 1,000 are recognized as occupational disease, despite of an asbestos ban in 1993^6,7^.

The so-called secondary prevention aims at an early detection of cancer in high-risk groups like occupationally asbestos-exposed individuals to improve therapy options. The diagnosis of MM can be difficult and is based on examination of tissue or cellular material by an experienced pathologist, generally supported by a panel of immunohistochemical markers^8^. For screening of an at-risk population, only less invasive methods like imaging can be employed. Unfortunately, imaging methods for MM screening are currently not available^9,10^. The non-circular tumor growth pattern of MM and the differentiation of pleural changes, especially between early MM and plaques, is challenging when interpreting computed tomography (CT) scans^11^. Moreover, a benefit from annual scans by CT, as shown for lung cancer, or other imaging methods has not been demonstrated yet for MM^12,13^. An alternative method could be the detection of blood-based biomarkers as recommended in the report on the updated Helsinki criteria^14^. Recently, Cohen *et al*. described a promising multi-analyte blood test for a number of different cancers^15^. Such tests are also known as ‘liquid biopsies’, a term originally coined for the analysis of circulating tumor cells but increasingly used for circulating DNA, RNA, and other markers as well^16^. Many blood-based tumor markers have also been described for MM but most of them only in relatively small case-control studies with cross-sectional design or without verification in an independent study population. Except for mesothelin, none has been evaluated in a sufficiently large prospective cohort study with serial prediagnostic samples of MM cases so far^17–22^. Up to this point, the performance of single markers in prediagnostic samples of cancer patients remains disappointing and might have discouraged the evaluation of more marker candidates^18,23,24^. Particularly the high costs and long duration of prospective studies may have contributed to the dearth of validated biomarkers for early detection of MM^25^.

In previous studies, we have established an assay for the protein marker calretinin and confirmed the results in independent groups of patients from three continents, using a conventional case-control design^26–28^. These studies usually enroll patients with more advanced tumor stages and lack prediagnostic samples to assess the performance of markers to detect tumors prior to the occurrence of clinical symptoms^25^.

We therefore established a high-risk cohort of former asbestos workers with annual examinations and blood sampling over a period of ten years. Prediagnostic samples of incident cases were used to evaluate the biomarkers calretinin and mesothelin in a nested case-control design.

## Results

### Characteristics of the study groups

**Figure 1:**
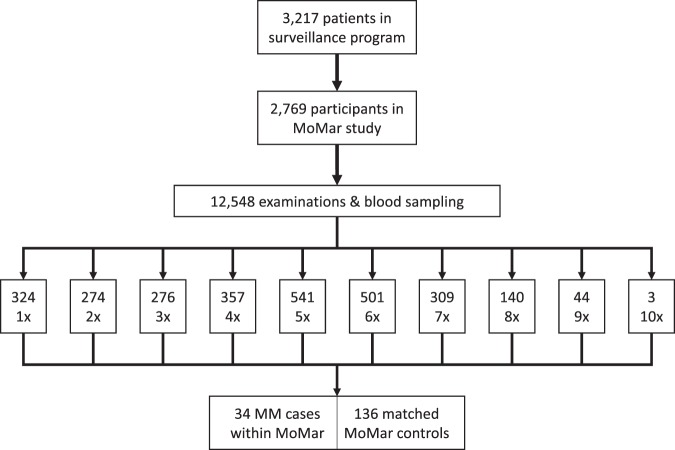
Study design and characteristics of the MoMar cohort. 2,769 asbestos-exposed workers with non-malignant asbestos-associated diseases participated in the study. Over a period of ten years, patients were invited annually for a medical exam and blood sampling. The patients participated up to ten times in the serial exams. Samples were stored in a biobank. At the completion of recruitment, incident MM cases were matched 1:4 to controls from the cohort, using a nested design, and biomarkers were determined.

During a period of about ten years 2,769 participants of a surveillance program of the German Social Accident Insurance for asbestos workers were recruited at 26 medical centers throughout Germany. The participants of the MoMar (‘Molecular Markers’) cohort took part up to ten times in annual examinations, which included the collection of prediagnostic blood samples. The response rate was 86.1% at initial recruitment but not everybody continued participation or attended annually. In total, 12,548 serial blood samples could be collected and archived. Until December 2017, 34 cases of MM were diagnosed. The median age of the male cases was 74 years (range, 64–83 years). An individual matching of 1:4 by age of patient and time of blood sampling resulted in 136 cancer-free controls (median age 74 years, range, 61–84 years). All cases and controls had benign asbestos-related diseases (asbestosis, pleural plaques, pleural thickening, and/or pleural fibrosis). The distribution of subtypes included 61.3% epithelioid, 19.4% biphasic, and 9.7% sarcomatoid MM; for 9.7% of the cases the subtype was not known. Because for three MM cases only a sample at the day of diagnosis (or less than one month before) was available, those cases (#3, #19, and #29) were excluded from further analysis, resulting in 31 eligible cases for the analysis of the performance of markers for an early detection of MM. For the remaining 31 cases, the arithmetic mean of the difference between sample collection and diagnosis was 11.1 months, the median 8.6 months (range, 1.3–44.4 months). The smoking status was not associated with the case-control status (former versus never smokers OR 0.92, 95% CI 0.39–2.28; current versus never smokers OR 0.95, 95% CI 0.22–4.16) and, therefore, was not adjusted for in the evaluation of the markers. Characteristics of the study groups are depicted in Table 1 and Supplementary Table 1. The study design is outlined in Fig. 1.

**Table 1:** Characteristics of malignant mesothelioma (MM) cases and matched controls in the nested case-control study of the MoMar cohort.

Characteristics	MM cases	Matched controls	P-value for group differences
Total	31*	136	
**Year of blood drawing**			
Median (Range)	2013 (2008–2015)	2013 (2009–2016)	
**Age at blood drawing [years]**			
Median (Range)	74 (64–83)	74 (61–84)	
Age groups, n (%)			1.0**
61–65	3 (9.7)	12 (8.8)	
66–70	3 (9.7)	16 (11.8)	
71–75	15 (48.4)	64 (47.1)	
76–80	9 (29.0)	40 (29.4)	
81–85	1 (3.2)	4 (2.9)	
**Time to diagnosis [months]**			
Median (Range)	8 (1–44)		
Interval, n (%)			
1–6	9 (29.0)		
7–12	13 (41.9)		
≥13	9 (29.0)		
**MM subtype, n (%)**			
Epithelioid	19 (61.3)		
Sarcomatoid	3 (9.7)		
Biphasic	6 (19.4)		
Not specified	3 (9.7)		
Smoking status, n (%)			0.95**
Never smokers	8 (25.8)	33 (24.3)	
Former smokers	20 (64.5)	90 (66.2)	
Current smokers	3 (9.7)	13 (9.6)	
Calretinin [ng/ml] Median (IQR)	0.36 (0.21–0.60)	0.19 (0.12–0.28)	<0.0001***
Mesothelin [nmol/l] Median (IQR)	1.35 (0.89–2.10)	0.96 (0.74–1.41)	0.0043***

All cases and controls were males. IQR, interquartile range; controls, never cancer-diseased participants with benign asbestos-related diseases. *Three of the 34 MM cases were excluded from analysis because the time between sampling and diagnosis was <1 month. **P-value obtained from Fisher’s exact test. ***P-value obtained from two-sided two-sample Wilcoxon rank-sum test.

### Performance of the biomarkers

**Figure 2:**
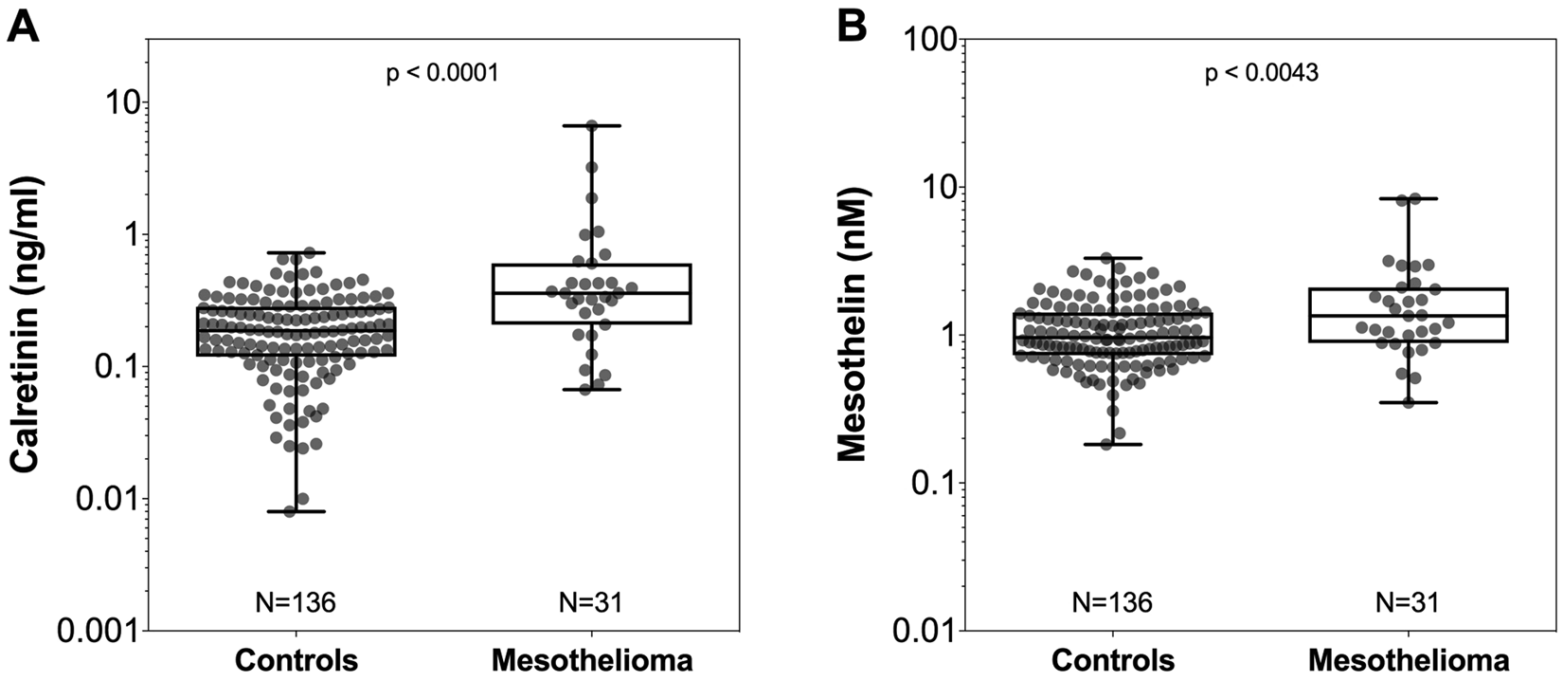
Distribution of marker concentrations in cases and controls. Concentrations of (**A**) calretinin (ng/ml) and (**B**) mesothelin (nM) in prediagnostic plasma samples of 31 MM cases and 136 matched controls. P-values were obtained from the two-sided Wilcoxon rank-sum test. Whiskers represent the minimum and maximum.

For each MM patient we used the last plasma sample available before diagnosis. If the last sampling was less than one month before diagnosis, the next available serial sample, usually from the sampling one year earlier, was taken. At first, we included all 31 patients with prediagnostic samples, ranging from about 1 to 44 months before diagnosis, for the assessment of the performance of the markers. The distribution of the plasma concentrations of calretinin and mesothelin in 31 cases and 136 controls are summarized in Table 1 and Fig. 2. The median concentration for calretinin was 0.359 ng/ml in MM cases and 0.187 ng/ml in controls. The difference between MM cases and controls was statistically significant (p < 0.0001). For mesothelin the median concentration was 1.349 nM in MM cases and 0.963 nM in controls (p = 0.0043).

**Figure 3:**
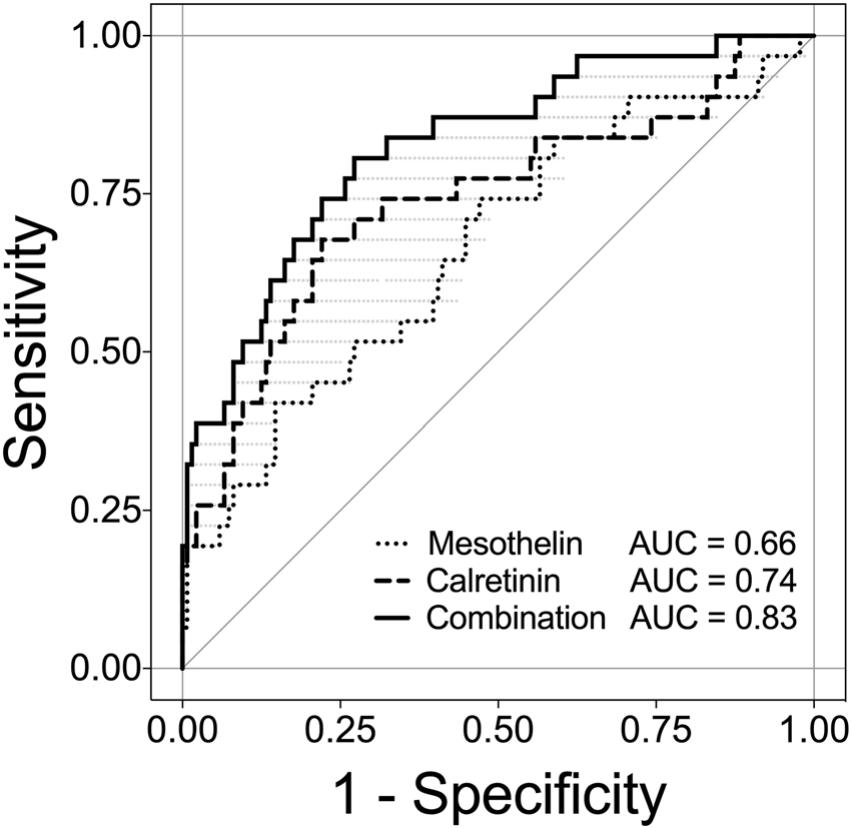
ROC analysis of calretinin, mesothelin, and the combination of both markers for the 1–44 months interval. ROC curves and AUC values (confidence intervals are listed in Table 3) of the individual and combined markers, based on 31 MM cases and 136 controls. All of the samples are included that have been obtained 1–44 months before diagnosis. The marker combination is based on a “best case” sequential combination.

The receiver operating characteristic (ROC) analysis of calretinin, mesothelin, and the “best case” scenario from sequential combination of both markers yielded an area under the curve (AUC) of 0.74 (95% CI, 0.63–0.85), 0.66 (95% CI, 0.55–0.78), and 0.83 (range, 0.65–0.83), respectively (Fig. 3). The sensitivities of calretinin and mesothelin for different pre-defined specificities and corresponding marker cutoffs are listed in Table 2. For example, at a pre-defined specificity of 98%, calretinin reached a sensitivity of 26% and mesothelin a sensitivity of 19%.

**Table 2:** Performance of calretinin and mesothelin for the detection of malignant mesothelioma based on 31 cases and 136 controls.

Specificity	Biomarker cutoff	Sensitivity	AUC (95% CI)
Calretinin	[ng/mL]		0.74 (0.63–0.85)
0.99	0.704	0.19	
0.98	0.604	0.26	
0.97	0.518	0.26	
0.96	0.507	0.26	
0.95	0.478	0.26	
0.90	0.393	0.42	
Mesothelin	[nmol/L]		0.66 (0.55–0.78)
0.99	2.910	0.19	
0.98	2.696	0.19	
0.97	2.629	0.19	
0.96	2.574	0.19	
0.95	2.314	0.19	
0.90	1.963	0.29	

Plasma samples were obtained 1–44 months before diagnosis. Performance measures are based on nonparametric ROC curves with the matched control group. AUC, area under the curve; CI, confidence interval.

### Biomarker performance depending on different time intervals

**Figure 4:**
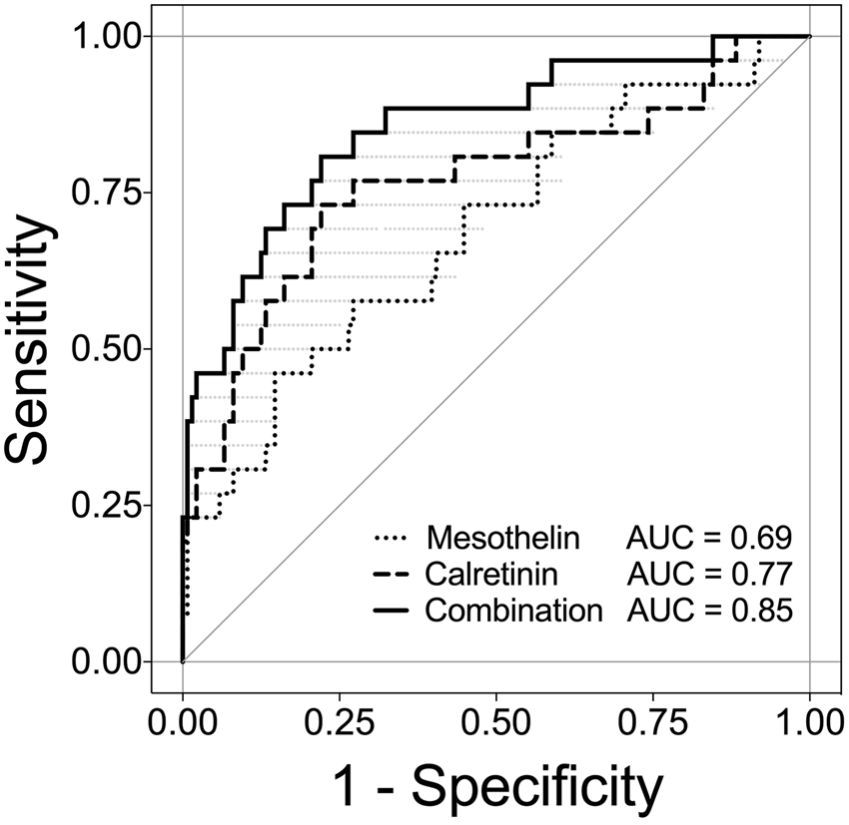
ROC analysis of calretinin, mesothelin, and the combination of both markers for the 1–15 months interval. ROC curves and AUC values (confidence intervals are listed in Table 3) of the individual and combined markers, based on 26 MM cases and 136 controls. Only samples are included that have been obtained 1–15 months before diagnosis. The marker combination is based on a “best case” sequential combination.

We also investigated the performance of the markers considering different screening intervals. It can be expected that, under realistic conditions, participants of a future screening program might sometimes skip an examination or might not always get an appointment in time – as seen with the current cohort. We therefore investigated the marker performance by time interval between last sampling before diagnosis and the date of diagnosis. Consequently, analyses were performed with smaller datasets containing all matched controls and only those MM patients with samples collected not more than 6, 9, 12, 15, or 24 months before diagnosis (Table 3). As an example, the ROC curves for the 15 months interval are shown in Fig. 4. The derived sensitivity for calretinin was 31% at a pre-set specificity of 98% and the sensitivity for mesothelin was 23% at a pre-set specificity of 99%. The sequential combination of both markers reached a sensitivity of 46% at a pre-set specificity of 98% (Table 3). Finally, we looked at the influence of the time interval on the effectiveness of the marker combination. The relative gain in sensitivity of the marker combination compared to calretinin alone increased with the length of the interval (Table 3).

**Table 3:** Performance of mesothelin, calretinin, and their combination in prediagnostic plasma samples depending on the time interval before diagnosis in which sampling took place.

Interval	Cases	Calretinin			Mesothelin			Sequential combination			
[Months]	[N]	AUC (95% CI)	Sens. [%]	Spec. [%]	AUC (95% CI)	Sens. [%]	Spec. [%]	AUC* (range)	Sens. [%]	Spec. [%]	Relative gain in sensitivity [%]
1–44	31	0.74 (0.63–0.85)	26	98	0.66 (0.55–0.78)	19	99	0.83 (0.65–0.83)	39	98	50
1–24	27	0.76 (0.64–0.87)	30	98	0.70 (0.58–0.81)	22	99	0.85 (0.68–0.85)	44	98	47
1–15	26	0.77 (0.65–0.88)	31	98	0.69 (0.57–0.81)	23	99	0.85 (0.68–0.85)	46	98	48
1–12	22	0.78 (0.65–0.90)	32	98	0.71 (0.59–0.82)	23	99	0.87 (0.68–0.87)	45	98	41
1–9	16	0.79 (0.65–0.94)	38	98	0.72 (0.58–0.85)	25	99	0.90 (0.70–0.90)	50	98	32
1–6	9	0.83 (0.65–1.00)	44	98	0.69 (0.49–0.88)	22	99	0.92 (0.69–0.92)	56	98	27

AUC, area under the curve; Sens., Sensitivity; Spec., Specificity; CI, confidence interval. *AUC calculation for the sequential combination is based on a “best case” scenario.

### Further evaluation of the marker combination

**Figure 5:**
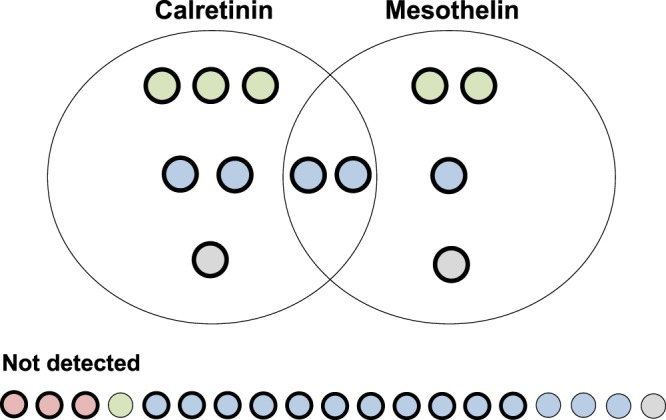
Venn diagram of all eligible MM cases of the cohort and correlation with marker results. A total of 12 MM tumors in 31 eligible cases were detected at a specificity of 98%. Calretinin detected eight and mesothelin six tumors, with two tumors detected by both markers. MM subtypes are indicated. The three sarcomatoid MM are labeled red, the six biphasic MM green, the 19 epithelioid MM blue, and the three MM without known histology grey. All cases within the 1–44 months interval between sample collection and diagnosis are shown, the cases within the 1–15 months interval are highlighted by a thick bordering.

The ROC analysis already indicated that calretinin and mesothelin are complementing each other. To better visualize the extent of complementation, a Venn diagram was drawn with all 31 eligible cases. The Venn diagram was based on a pre-defined specificity of 98% for each marker and included all cases that were correctly detected by either or both assays. Only two cases were detected jointly by both markers while the calretinin assay added six and the mesothelin assay another four positive cases (Fig. 5). The histology of MM can have an influence on the marker performance^27,29^. Therefore, MM subtypes are indicated in the Venn diagram. The three sarcomatoid MM of the MoMar cohort were neither detected by the calretinin nor the mesothelin assay. In contrast, five of the six biphasic cases were identified by at least one of the assays.

Individual measurements of the fiber concentrations or a job-exposure matrix were not available for this cohort. We therefore applied an expert rating to categorize recognized at-risk occupations into moderate, high, and very high exposure to asbestos (Supplementary Table S1) and tested the biomarker distributions by exposure level in controls (Supplementary Table S2). Both biomarkers were not associated with asbestos exposure (calretinin: p = 0.32; mesothelin: p = 0.57).

## Discussion

The goal of cancer screening is to facilitate early detection of tumors at a potentially curative stage and, ultimately, to reduce mortality. Treatment options are improved when the cancer has not yet spread and the patient’s overall condition is not a limiting factor. Currently, the prognosis for MM patients is still dismal, however, therapies are gradually improving and promising new approaches – like immunotherapy – are being investigated^30–33^. Without timely treatment the full potential of existing and future therapies may not be realized. Therefore, methods for early detection such as simple blood tests or imaging procedures are urgently needed.

The detection of cancer prior to the occurrence of clinical symptoms in at-risk populations can only be performed with non- or minimally-invasive methods, such as biomarkers in body fluids or with imaging^25^. Due to the growth pattern of MM imaging is challenging^9,11^, which strengthens the potential role of tumor markers. Biomarkers, owing to their noninvasive or minimally-invasive nature, do not constitute a burden for the patient like biopsies or radiation^25^. Blyuss *et al*. recently demonstrated in a longitudinal study that a panel of the biomarkers CA125, HE4, and glycodelin was able to detect ovarian cancer in prospectively collected samples up to one year before diagnosis^34^. Despite that numerous biomarkers to detect MM have been described before, except for mesothelin, none have been properly validated in a prospective cohort to prove their potential as markers for early detection^17–21^.

To our knowledge, this is the first study to evaluate the new plasma marker calretinin as well as its combination with the established marker mesothelin in a large prospective cohort study. Furthermore, we independently validated/confirmed the prediagnostic potential of mesothelin, previously evaluated by Creaney *et al*. in their cohort^18^. Very few cohorts with serial prediagnostic serum or plasma samples of asbestos-exposed workers exist worldwide^17,18,21^ because such a design is costly and time-consuming. We took advantage of an already existing surveillance program of secondary prevention in asbestos workers provided by the statutory accident insurances in Germany that allowed us to obtain serial prediagnostic blood samples of almost 2,800 subjects with a high response rate while limiting the costs associated with setting up a cohort *de novo*.

A limitation of our and many other prospective studies on rare diseases is the low number of incident cases during follow-up. We were able to include 31 of 34 cases that were diagnosed during a period of about ten years. More cases would have required the recruitment of substantially more subjects or a longer involvement of the participants. However, many of the former asbestos workers (up until the end of sample collection more than 1,000) had already reached a high age and were discontinuing their participation, or were deceased. The rareness of MM also limits sound results on factors like subtype or localization of the tumor.

Previous studies, using a conventional case-control design, have shown that sarcomatoid MM are less well detected by the mesothelin and basically not detected by the calretinin assay^27,29^. In accordance, the three sarcomatoid cases of our prospective cohort were detected by none of the two markers. Thus, the detection of sarcomatoid MM appears to remain a challenge for biomarkers. In comparison, most of the biphasic MM were identified. However, for both subtypes the number of cases was too low to draw a sound conclusion.

The study design is a nested case-control comparison as a pre-final step in tumor marker research prior to its approval for screening^25^. In this step, a positive test result does not initiate an invasive diagnostic work-up. Creaney *et al*. were the first to evaluate mesothelin using a nested case-control comparison based on the prospective Wittenoom cohort, determining a sensitivity of 25% at a specificity of 97%^18^. The study comprised 106 cases and 99 asbestos-exposed controls. The median of the sampling interval of the cases was 8 months before diagnosis (range, one week to 13 years), which is similar to ours (8.6 months) while in our study the range was one month to 3.7 years. We were able to independently confirm the results with small differences. Besides a variation by chance in such small case groups, the small differences may be attributed to the distribution of age, gender, and benign pathologies (asbestosis, pleural plaques, pleural thickening, etc.) in our cases and controls. For example, using patients with asbestosis as controls is likely more challenging for assay performance than asbestos-exposed controls without known respiratory diseases^27,35,36^.

A major challenge of introducing cancer screening into practice is a protocol that includes measures to limit false-positive results^37^. This implies the assessment of sensitivity conditional on a high specificity^25^. Of note, the described markers were studied regarding their diagnostic performance but not their prognostic properties. High specificities are intended to avoid unnecessary psychological burden and invasive diagnostic workup for the patients^25,37^. We therefore focused on high pre-defined specificities of the marker assays we studied. The sensitivity of the calretinin assay (26%) was similar to that obtained for mesothelin at pre-defined 98% specificity in the 1–44 months interval before diagnosis. Regarding the length of the time interval, we have tested several scenarios that might occur in future screening programs. In addition to the 44 months interval, we have analyzed the assay performance for an interval starting 6, 9, 12, 15, and 24 months before diagnosis. The performance of calretinin as well as the marker combination improved with decreasing time until diagnosis. The sensitivity of the marker combination reached 56% when samples were taken between 1 and 6 months before diagnosis. The smaller number of cases in these time intervals (just 9 cases in the 6-months interval), however, limits the statistical power. A likely scenario would be a screening interval of 12 months. In reality, delays in scheduling may increase an interval up to several months for an individual. We therefore deem an interval of 15 months as a realistic basis to judge the performance of the markers. Under that condition, 26 cases could be included in the ROC analysis, which resulted in a sensitivity of 46% conditional on a specificity of 98% for the marker combination. The corresponding cutoffs were 0.60 ng/ml for calretinin and 2.91 nM for mesothelin.

According to the model of Lin and Plevritis the growth of a tumor is exponential and depends on its volume doubling time^38^. If we assume that the concentration of released biomarkers is proportional to the size of a tumor, there might only be a narrow time window for screening of tumors with a high growth rate^25,38^. Blyuss *et al*., using prediagnostic samples of the PLCO cohort, have shown that early detection of ovarian cancer by biomarkers is most likely within one year before diagnosis^34^. Also, in previous cohort studies on MM with almost all samples available more than one year before diagnosis we and others did not see any mesothelin levels above the cutoff of 2.9 nM^17,39^. Similarly, we have seen a narrow window in another prospective cohort where bladder cancer markers reached clinical cut-offs more frequently in the year before diagnosis^40^. In contrast, an early detection of MM by biomarkers several years before diagnosis was reported for individual patients in smaller studies^20,41^. The current results would be in line with a narrow window of less than two years to capture a tumor prior to diagnosis with high specificity.

At first glance, the relatively low sensitivities of the individual markers may appear disappointing compared to the results obtained in classical case-control studies with patients in more advanced tumor stages. However, considering the above model, prediagnostic samples are expected to release less molecules into the bloodstream. To overcome this problem, two or more markers can be combined in order to increase the sensitivity. This has been shown for MM by several groups using conventional case-control comparisons but has not been shown yet for a longitudinal study design^27,39,42–47^. Preconditions for a successful combination are a high specificity of all individual markers to limit a possible loss of the overall specificity, and sufficient complementation to allow for a gain in sensitivity.

Previously, we have tested a combination of calretinin and mesothelin using case-control studies that included symptomatic MM patients, thus with tumors at later stages of development^27,28^. Comparing the more sensitive of the two markers with their combination, the increase in sensitivity was relatively small in the Australian/German (14%) and the Mexican study groups (about 13%). Interestingly, we saw a more pronounced complementation of both markers in the prediagnostic samples of the MoMar cohort, resulting in an increase ranging from 27% (1–6 months interval) to 50% (1–44 months interval). A similar effect has been observed with a marker panel for the detection of pancreatic cancer using prediagnostic samples from a large cohort^48^. A possible explanation could be that the biomarker spectrum of an individual tumor is more distinct at earlier stages, whereas at later stages of development – when tumor heterogeneity is increasing^49–51^ – the spectrum of molecular markers in a growing tumor can broaden with time and cause more overlap with other tumors. Thus, the likelihood of expressing calretinin and mesothelin simultaneously would increase with the growth and stage of the tumor. The notion that complementation of biomarkers for early detection could benefit indirectly from the molecular evolution of cancer may be worth pursuing.

Another way to improve the performance of assays is by controlling for potential confounders. We and others have already investigated possible influencing factors of mesothelin and calretinin^52–57^. Including age, kidney function, and other clinically relevant factors in statistical models may further decrease the number of false-positive results but has to await a more in-depth analysis of the MoMar cohort. Because we matched cases and controls by age, adjustment for this parameter did not lead to improvements. Impaired filtration by the kidneys can lead to an accumulation of proteins with lower molecular weight like soluble mesothelin-related peptides (SMRP)^53^. Because the cutoff we used for mesothelin was relatively high compared to other studies this confounder may have had less influence in our study^54–56^. Smoking was not a predictor of elevated marker levels as has been shown in previous studies^26,54,55^. Likewise, as observed before in another cohort^18^ asbestos exposure appeared not to be an influencing factor. Of note, duration of exposure and individual historical data on fiber years are lacking in this study, and a comprehensive exposure assessment is usually not feasible in clinical settings^25^. Preanalytical factors like sample storage can influence the performance of markers. However, calretinin and mesothelin are stable regarding storage time and freeze/thaw cycles^26,27,52,58^.

In conclusion, we have shown for the first time that calretinin is able to detect MM with a high specificity in prediagnostic plasma samples up to about a year before diagnosis. The moderate sensitivity is markedly increased by combination with mesothelin. The strength of the study is based on the use of a prospective cohort of workers with benign asbestos-related diseases but limited by the number of incident cases. While the performance of the markers and their combination decreases with samples more distant to diagnosis, the relative gain in sensitivity through marker complementation appears to increase with the length of the time interval before diagnosis. This is an interesting effect that can be further investigated with other marker combinations and other cancers. The encouraging results obtained by measurement of calretinin and mesothelin in ‘prediagnostic liquid biopsies’, however, have to be confirmed by testing the marker combination in an independent cohort with serial prediagnostic samples. In parallel, the biobank of the MoMar cohort will serve as a valuable resource for evaluation and validation of additional markers to increase the sensitivity of the MM marker panel. Calretinin and mesothelin might be able to detect MM at earlier stages to improve therapy options, however, a possible reduction in mortality by early detection can only be assessed when better therapies for MM become available.

## Methods

### Recruitment and study population

The participants of the MoMar cohort were former asbestos workers who had a recognized occupational disease (asbestosis and/or other (nonmalignant) pleural diseases caused by asbestos) and took part in the program of secondary prevention regularly offered by the statutory accident insurances. For the MoMar study, the regular examinations were extended by the voluntary donation of a blood sample and a short questionnaire that documented smoking status and at-risk occupations in addition to other data. We applied an expert rating (B.P., M.L.) based on these occupations to assign very high, high, and moderate exposure to asbestos blinded by case-control status. The participants were recruited from November 2008 until February 2018 at 26 medical centers throughout Germany. The medical centers were either clinics or private practices of pulmonologists. During follow-up, the status of the participants (malignant disease or death) was checked in regular intervals of one year and information regarding diagnosis (histology, date of diagnosis, date of death, etc.) was obtained through the statuary accident insurances.

All participants of the MoMar cohort gave written informed consent. The methods were carried out in accordance with the relevant guidelines and regulations. The study was approved by the ethics committee of the Ruhr-University Bochum (reference number 3217-08).

### Blood collection

Blood was collected into 9.0 ml S-Monovettes EDTA gel tubes (Sarstedt, Nümbrecht, Germany). After separation by centrifugation (2,000 × g for 10 minutes at room temperature within 30 minutes after collection), plasma and cell pellet were frozen immediately and temporarily stored on-site at −20 °C. Samples were regularly collected and transported to the IPA, aliquoted, and stored at −80 °C in the IPA biobank until use.

### Determination of calretinin

Concentrations of calretinin in plasma samples were determined by enzyme-linked immunosorbent assays (ELISA), using the Calretinin ELISA kit by DLD Diagnostika GmbH (Hamburg, Germany) according to the manufacturer’s instructions. This assay is based on the same antibodies as used in the previously described version of the calretinin ELISA^26–28,55^. During the procedure, all reagents and samples were equilibrated to 22 °C and the incubations were performed at 22 °C. Plasma samples (30 µl) were diluted 1:5 in the provided dilution buffer. The diluted samples were determined in duplicate.

Optical densities were measured on a SpectraMax 384 plus plate reader (Molecular Devices, Sunnyvale, CA, USA). The standard curve was obtained by four-parameter curve fitting using SoftMax Pro 4.7.1 from Molecular Devices.

### Determination of mesothelin

Concentrations of mesothelin (SMRP, soluble mesothelin-related peptides) in plasma samples were determined using the ELISA kit MESOMARK by Fujirebio Diagnostics, Inc. (Malvern, PA, USA) according to the manufacturer’s instructions with modifications as described before^58^.

### Statistical analysis

The 34 male cases were matched 1:4 to never cancer-diseased male participants by patient age in 5-year bins and sample age in 7-months bins, resulting in 136 controls. Calretinin and mesothelin concentrations were presented as box plots with median and interquartile range (IQR), overlaid with dot plots. Whiskers represent minimum and maximum. Less than 8% of calretinin values were below the limit of detection and therefore not specifically addressed. The two-sample Wilcoxon rank-sum test was applied to compare the distribution of calretinin and mesothelin values between MM cases and controls. Fisher’s exact test was used to compare age groups and smoking status between both groups.

Biomarker classification performance was determined by ROC curve with the AUC estimated to assess a marker’s sensitivity for varying values of specificity. 95% Wald confidence intervals (CI) were calculated for the AUCs. For the sequential combination of both markers, first calretinin was used for classification. Afterwards, mesothelin was examined for calretinin-negative subjects only. This procedure was repeated for all possible cutpoints to calculate the related sensitivities and specificities resulting in a point cloud instead of a traditional ROC curve. Therefore, the AUC for this combination was calculated as descriptive measure for the “best case” scenario, so that the points with highest specificities and sensitivities were considered. The related intervals show minimum and maximum obtainable AUCs. Statistical analyses were performed using SAS/STAT and SAS/IML software, version 9.4 (SAS Institute Inc., Cary, NC, USA) and GraphPad Prism version 7.04 (GraphPad Software, La Jolla California, USA) was used for generating graphs.

## Electronic supplementary material

Supplementary Table S1

Supplementary Table S2

## Data Availability

All original data analyzed during this study are included in this published article (and its Supplementary Information files).
