# Supplementary material for: Prediagnostic detection of mesothelioma by circulating calretinin and mesothelin – a case-control comparison nested into a prospective cohort of asbestos-exposed workers
Source: Sci Rep. 2018 Sep 25;8:14321. doi: 10.1038/s41598-018-32315-3 (PMC6156219; doi:10.1038/s41598-018-32315-3)
Supplement: Supplementary file 2 — Supplementary Table S2 [file 41598_2018_32315_MOESM2_ESM.docx]

**Prediagnostic detection of mesothelioma by circulating calretinin and mesothelin – a case-control comparison nested into a prospective cohort of asbestos-exposed workers**

Georg Johnen^1^*, Katarzyna Burek^1^, Irina Raiko^1^, Katharina Wichert^1^, Beate Pesch^1^, Daniel G. Weber^1^, Martin Lehnert^1^, Swaantje Casjens^1^, Olaf Hagemeyer^1^, Dirk Taeger^1^, Thomas Brüning^1^ & MoMar Study Group^1^

^1^Institute for Prevention and Occupational Medicine of the German Social Accident Insurance (IPA), Institute of the Ruhr University Bochum, Bochum, Germany

*Correspondence and requests for materials should be addressed to G.J. (e-mail: johnen@ipa-dguv.de)

| Asbestos exposure | N |  | Calretinin [ng/mL] | |  | Mesothelin [nM] | |
| --- | --- | --- | --- | --- | --- | --- | --- |
|  |  |  | Median | P-value* |  | Median | P-value* |
| Very high | 48 |  | 0.209 |  |  | 0.986 |  |
| High | 68 |  | 0.175 |  |  | 0.907 |  |
| Moderate | 19 |  | 0.175 |  |  | 1.066 |  |
|  |  |  |  | 0.32 |  |  | 0.57 |

**Supplementary Table S2.** **Marker concentrations by asbestos exposure in 135 controls**

*Kruskal-Wallis test
